# Comparative Transcriptome Analysis Reveals the Mechanisms Underlying Differences in Salt Tolerance Between *indica* and *japonica* Rice at Seedling Stage

**DOI:** 10.3389/fpls.2021.725436

**Published:** 2021-10-27

**Authors:** Weilong Kong, Tong Sun, Chenhao Zhang, Xiaoxiao Deng, Yangsheng Li

**Affiliations:** ^1^State Key Laboratory of Hybrid Rice, College of Life Sciences, Wuhan University, Wuhan, China; ^2^Shenzhen Branch, Guangdong Laboratory for Lingnan Modern Agriculture, Genome Analysis Laboratory of the Ministry of Agriculture, Agricultural Genomics Institute at Shenzhen, Chinese Academy of Agricultural Sciences, Shenzhen, China

**Keywords:** salt stress, comparative transcriptome, Meta-QTLs mapping, rice (*Oryza sativa* L.), seedling stage

## Abstract

Screening and breeding more salt-tolerant varieties is an effective way to deal with the global reduction in rice (*Oryza sativa* L.) yield caused by salt stress. However, the molecular mechanism underlying differences in salt tolerance between varieties, especially between the subspecies, is still unclear. We herein performed a comparative transcriptomic analysis under salt stress in contrasting two rice genotypes, namely RPY geng (*japonica*, tolerant variety) and Luohui 9 (named as Chao 2R in this study, *indica*, susceptible variety). 7208 and 3874 differentially expressed genes (DEGs) were identified under salt stress in Chao 2R and RPY geng, separately. Of them, 2714 DEGs were co-expressed in both genotypes, while 4494 and 1190 DEGs were specifically up/down-regulated in Chao 2R and RPY geng, respectively. Gene ontology (GO) analysis results provided a more reasonable explanation for the salt tolerance difference between the two genotypes. The expression of normal life process genes in Chao 2R were severely affected under salt stress, but RPY geng regulated the expression of multiple stress-related genes to adapt to the same intensity of salt stress, such as secondary metabolic process (GO:0019748), oxidation-reduction process (GO:0009067), etc. Furthermore, we highlighted important pathways and transcription factors (TFs) related to salt tolerance in RPY geng specific DEGs sets based on MapMan annotation and TF identification. Through Meta-QTLs mapping and homologous analysis, we screened out 18 salt stress-related candidate genes (RPY geng specific DEGs) in 15 Meta-QTLs. Our findings not only offer new insights into the difference in salt stress tolerance between the rice subspecies but also provide critical target genes to facilitate gene editing to enhance salt stress tolerance in rice.

## Introduction

Rice (*Oryza sativa* L.) is one of the main food crops in the world and feeds nearly half of the world’s population (Parihar et al., 2015; Kong et al., 2019b). Soil salinity affects the growth and yield of rice in approximately one-third of the rice-growing land in the world (Hakim et al., 2014; Wang et al., 2018). Therefore, improving the salt tolerance of rice is essential to ensure food security for billions of people around the world. Plant salt stress tolerance is a very complex mechanism involving ion-homeostasis, ion-compartmentalization, osmoprotectant biosynthesis, and antioxidative defense (Ganie et al., 2019, 2021). So far, intensive efforts have been devoted to the dissection of genetic regulation of rice salt tolerance through reverse and forward genetic approaches (Ganie et al., 2016).

The salt stress tolerance of rice is related to the genotype, and different genotypes show discrepant salt stress tolerance (Ganie et al., 2016; Mansuri et al., 2019). Hundreds of salt stress-related QTLs have been identified among different rice populations to date (Kong et al., 2019b; Mansuri et al., 2020). However, only four salt stress-related genes have been cloned and experimentally verified in rice, namely, *SKC1* (Lin et al., 2004; Hauser and Horie, 2010), *HST1* (Takagi et al., 2015), *OsHAK21* (He et al., 2019), and *OsSTLK* (Lin et al., 2020). Candidate genes in most QTLs reported so far are unknown due to the too large mapping interval. Meta-QTLs analysis can effectively narrow the mapping interval of QTLs to achieve reliable prediction of candidate genes by integrating the QTLs results from a large number of studies (Kong et al., 2020). 11 salt stress-related Meta-QTLs were reported *via* integrating QTL information from 12 studies (Islam et al., 2019). Recently, Mansuri et al. (2020) identified 46 Meta-QTLs of salt stress-related traits, including salinity tolerance score, shoot potassium concentration, shoot sodium concentration, chlorophyll content, shoot dry weight trait, etc., (Mansuri et al., 2020).

On the other hand, RNA-seq was utilized to detect genome-wide gene expression changes under salt stress and predict potential molecular networks in rice (Kong et al., 2019b; Mansuri et al., 2019, 2020). Many differentially expressed genes (DEGs) have been identified among the contrasting samples through RNA-sequencing analysis. For example, 995 and 1052 DEGs were identified in the shoot and root of Nipponbare (*japonica*) (Mizuno et al., 2010). Wang et al. (2018) conducted comparative transcriptome analysis between salt-tolerant and salt-sensitive genotypes of *indica* rice and reported 5,273 salt-responsive DEGs (286 DEGs only in the tolerant genotype) at the seedling stage (Wang et al., 2018). Another study compared transcriptome profiles of FL478 and its sensitive parent (IR29) and revealed 1063 DEGs were co-expressed in both FL478 and IR29 (Mansuri et al., 2019). It is well known that there are obvious differences in important agronomic traits and biotic/abiotic stresses between *indica* and *japonica* rice, and their hybrid progeny and derived lines often show obvious better-parent advantages in terms of yield and stress resistance (Yuan, 1994; Bian et al., 2010). *Indica* and *japonica* rice also have significant differences in abiotic stress resistance, including cold stress and salt stress (Yang et al., 2015). However, the molecular mechanism, key genes, and regulatory network of the difference in salt tolerance between *indica* and *japonica* rice are still unclear. In this study, we explored the genome-wide expression differences between RPY geng (*japonica*) and Chao 2R (*indica*) using RNA-seq technology. The results of integrating DEGs and Meta-QTLs highlighted the key salt stress-related genes and possible regulatory networks. Our finding not only provided a better insight into the difference in salt tolerance between *indica* and *japonica* rice but also contributed to potential gene targets for breeding better salt-tolerant varieties of *indica*-*japonica* hybrids.

## Materials and Methods

### Plant Materials and Treatments

Rice seedlings were grown in 96-well PCR plates with Yoshida solution (Coolaber, Beijing, China) replaced every 2 days, 26°C, and a 16/8 h light/dark photoperiod, 60% relative humidity in plant growth incubators (ZSX1500GS, Jingshen Instrument, Shanghai, China) for 14 days (Kong et al., 2020). Fourteen-day-old seedlings of RPY geng and Chao 2R were changed into 100 mM NaCl Yoshida solution for salt stress treatment (Supplementary Figure 1). Then, we sampled root tissues at 0-day (0d), 3-day (3d), and 7-day (7d), respectively. Three independent biological replicates were prepared for each treatment/control group, and at least 30 seedlings with uniform growth were sampled for each biological replicate. After all samples were collected and immediately stored in liquid nitrogen for the next step of RNA extraction.

To evaluate salt stress tolerance of RPY geng and Chao 2R, 14-day-old seedlings were treated with 125 mM NaCl Yoshida solution for 7 days to simulate salt stress. After salt stress treatment, the seedlings were grown in Yoshida solution for 7 days and the survival rate of the seedlings were calculated. 30–45 seedlings were used for each treatment and all treatments were placed randomly with three replicates. The final survival rate of rice seedlings came from the average of the survival rates of three replicates.

### RNA-Seq Sequencing and Transcriptome Analysis

RNA extraction and cDNA library construction of all tested samples were conducted by Biomarker Technologies (Beijing, China) according to the standard procedures. All cDNA libraries were sequenced on an Illumina sequencing platform (HiSeq 2500) by paired-end (2 bp × 150 bp) method. After the raw reads were filtered by Trimmomatic (Bolger et al., 2014), a total of 116.45 Gb clean reads were generated, and the percentage of Q30 bases in each sample was not less than 92.38% (Supplementary Table 1). Clean reads were mapped to RPY geng genome (assembled by our team, unpublished data) using Hisat2 software and quantified the mapped reads using StringTie software (Pertea et al., 2016). Gene expression levels were estimated by fragments per kilobase of transcript per million fragments mapped reads (FPKM) method. DESeq2 was used to identify DEGs with these limits: False Discovery Rate (FDR) < 0.01 and Fold Change ≥2.

### Differential Gene Annotation and Transcription Factor Identification

Gene ontology (GO) enrichment of DEGs was performed with GOseq R package, and GO terms with KS < 0.01 were considered to be significantly enriched (Young et al., 2010). KEGG pathway enrichment of DEGs was performed by KOBAS (Mao et al., 2005), and the pathway with *P*-value < 0.05 was considered to be significantly enriched.

MapMan annotation of RPY geng specific DEGs was conducted in the online annotation website^1^ with default parameters (Kong et al., 2020). iTAK software (Jin et al., 2017) was used^2^ to identify transcription factors (TFs) with default parameters.

### Meta-QTLs Mapping of Differentially Expressed Genes

Meta-QTLs were obtained from Mansuri et al. (2020) study. The physical locations of Meta-QTLs in IRGSP-1.0 genome were obtained by blasting SSR markers in the online blast tool^3^ of RAP-DB website (markers with a 100% match rate and the unique location in RAP-DB genome). IDs in RPY geng genome of DEGs were converted to IRGSP-1.0′ IDs through blastn with parameters: -max_target_seqs 1 -evalue 1e-6 -perc_identity 95. To ensure the accuracy of results, DEGs were further filtered based on the chromosome position and DEGs that were not on the same chromosome between RPY geng ID and IRGSP-1.0 ID were filtered out.

### RT-PCR Verification of RNA-Seq Results

In this study, five randomly selected candidate genes for DEGs were used for RT-PCR analysis. The specific primers for these five genes and actin gene (an internal control) were designed by Primer 5.0 (Supplementary Table 2). The qRT-PCR reaction (10 μL) was formulated using the 2 X SYBR Green qPCR Master Mix (US Everbright^®^Inc., Suzhou, China). All qRT-PCRs were carried out on a CFX96 Touch^TM^ Real-Time PCR Detection System (Bio-Rad, Hercules, CA, United States). Three biological replicates (from three independent RNA samples) were used for qRT-PCRs. For each biological replicate, three technical replicates were also conducted. The average threshold cycle (Ct) from three biological replicates was employed to calculate the gene expression fold change by the 2^–ΔΔ*CT*^ method (Kong et al., 2019a,b).

## Results

### RPY Geng Is More Tolerant to Salt Stress Than Chao 2R at Seedling Stage

**FIGURE 1:**
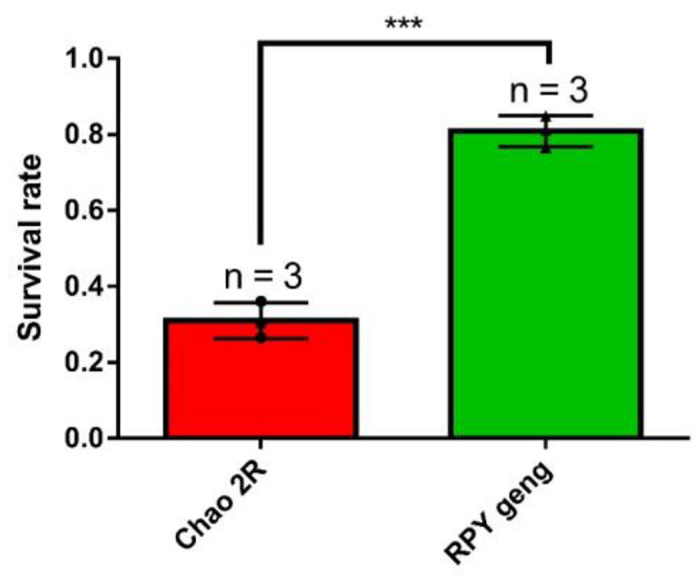
The final survival rate of rice seedlings (Chao 2R and RPY geng) after 7 days of salt stress treatment and 7 days of recovery from three independent replicates. *** represented *P* value < 0.001. The bar value was generated from three independent biological replicates. Survival rate = number of surviving seedlings/total number of seedlings in each biological replicate.

To characterize the difference in salt stress resistance between these two subspecies, we calculated the final survival rate of Chao 2R and RPY geng after salt treatment. We found that RPY geng had a higher survival rate than Chao 2R (81% vs. 31%) (Figure 1).

### RNA-Sequencing and Differentially Expressed Genes Results

**FIGURE 2:**
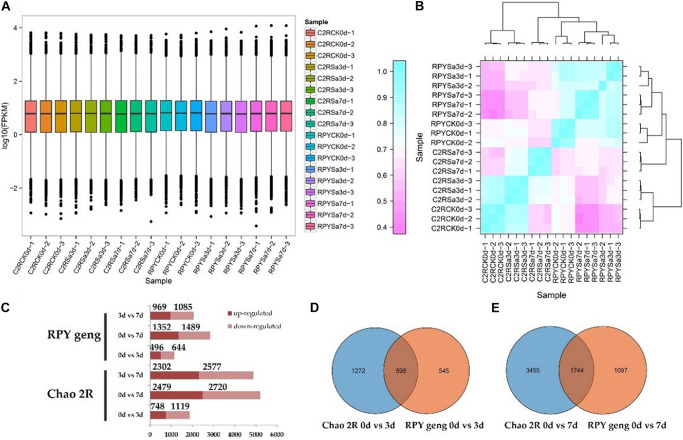
Sequencing data and differentially expressed genes (DEGs) results. **(A)** FPKM box diagram of all tested samples. **(B)** Heatmap of Pearson correlation coefficient for all tested samples. **(C)** Statistics results of DEGs in all comparison combinations. **(D,E)** The venn analysis of DEGs between Chao 2R and RPY geng in 3 or 7 days after stress treatment.

RNA-sequencing was performed on 18 samples (Supplementary Figure 1), and sequencing data for each sample exceeded 6G with high mapping rates (Supplementary Table 1). The overall gene expression levels of the three biological replicates of each treatment were similar (Figure 2A) and the three biological replicates were clustered together with the Pearson correlation coefficient approaching 1 (Figure 2B).

The results of DEGs analysis in RPY geng showed that there were 496 up-regulated and 644 down-regulated genes in 0d vs. 3d, 1352 up-regulated genes and 1489 down-regulated genes in 0d vs. 7d, and 969 up-regulated genes and 1085 down-regulated genes in 3d vs. 7d, respectively. However, Chao 2R had more DEGs than RPY geng in every comparison (Figure 2C). At 0d vs. 3d, RPY geng and Chao 2R shared 595 DEGs, at 0d vs. 7d, RPY geng and Chao 2R co-expressed 1744 DEGs (Figures 2D,E). Both RPY geng and Chao 2R had more DEGs in 0d vs. 7d than 0d vs. 3d, which indicated that more genes changed significantly the transcription levels with the extension of the salt stress time. The total number of DEGs in Chao 2R (7208) was significantly greater than that of RPY geng (3874), suggesting that the transcriptome of Chao 2R was more affected to salt stress, which along with the survival data indicated that Chao 2R was more sensitive to salt stress.

### Enrichment and Annotation of Differentially Expressed Genes

**FIGURE 3:**
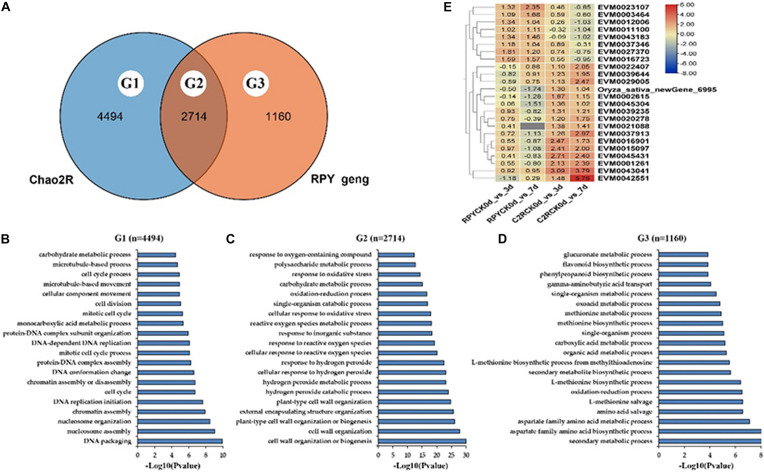
Go annotation and heatmap of DEGs. **(A)** Venn analysis of all DEGs between Chao 2R and RPY geng. **(B)** Top 20 Gene ontology (GO) terms of Chao-2R specific DEGs. **(C)** Top 20 GO terms of common DEGs in Chao 2R and RPY geng. **(D)** Top 20 GO terms of RPY geng specific DEGs. **(E)** Heatmap of DEGs with the opposite expressed profiles in two subspecies. RPY means RPY geng, C2R means Chao 2R, 0d, 3d, and 7d means control, 3rd day after treatment, and 7th day after treatment in panel **(B)**.

In total there were 2714 DEGs (G2) shared by Chao 2R and RPY geng, 4494 DEGs (G1) unique to Chao 2R and 1160 DEGs (G3) unique to RPY geng (Figure 3A). The top 20 GO terms of G1, G2, G3 were compared in Figures 3B–D. G2 were significantly enriched in well-known GO terms related to abiotic stresses, such as plant-type cell wall organization (GO:0042744), hydrogen peroxide metabolic process (GO:0042743), response to hydrogen peroxide (GO:0042542), and so on (Figure 3C). As expected, G1 and G3 were enriched in completely different GO terms. The GO terms of G3 were related to various stress-related processes (Figure 3D), while GO terms of G1 involved the necessary life activity pathways, namely, cell division (GO: 0051301), cell cycle process (GO:0022402), and etc., (Figure 3B). The above results indicated that under the same intensity of salt stress, the normal life process of Chao 2R were severely affected, and there were many DEGs in RPY geng that played important roles in salt stress tolerance. Therefore, we then focused on analyzing DEGs in G2 and G3. In G2, 24 DEGs showed the opposite gene expression profiles in Chao 2R and RPY geng (Figure 3E). We speculated that these DEGs may be related to the difference in salt stress tolerance between RPY geng and Chao 2R (Supplementary Table 3).

### Key Salt Stress Responsive Genes and Pathways in RPY Geng

**FIGURE 4:**
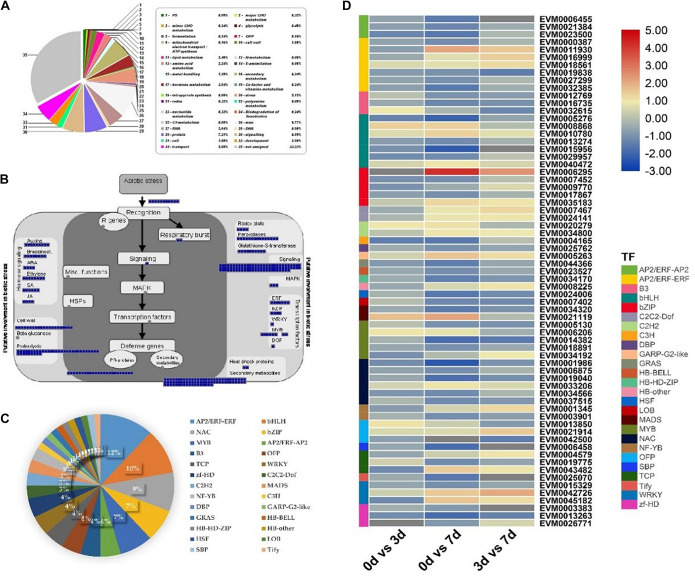
MapMan annotations and gene expression analysis of RPY geng specific DEGs (G2 + G3). **(A)** Functional categories of DEGs based on MapMan annotations. **(B)** The MapMan biotic stress overview of RPY geng specific DEGs. **(C)** The transcription factors (TFs) of RPY geng specific DEGs. **(D)** Gene expression profiles of TF genes. Blue boxes represented genes were differentially expressed on day 3 or/and on day 7 after salt stress treatment in panel **(B)**. The percentage of different TFs in the total TFs was represented by pie chart in panel **(C)**. The high-resolution of this figure was provided in Supplementary Figure 2.

Next, DEGs (G2 + G3) were further analyzed to explore the regulatory network of salt tolerance in RPY geng. The MapMan results showed that, RPY geng specific DEGs were mainly enriched in secondary metabolism, stress, signaling, transport, hormone metabolism, and other pathways in addition to essential cellular component pathways (protein, RNA, etc.) (Figure 4A). These DEGs were involved in many aspects of plant response to biotic stress, including signaling, mitogen activated protein kinases (MAPK) pathway, secondary metabolites, plant hormones (such as auxins, brassinosteroids, ABA, ethylene, SA, JA), and various functional proteins (Figure 4B). In addition, 26 types of TFs might also played an important role in salt stress tolerance, including AP2/ERF-ERF, bHLH, NAC, bZIP, MYB, and other TFs (Figure 4C). Under salt stress, the gene expression profiles of these TF genes were obviously differentiated with different direction, even within the same gene family, i.e., within AP2/ERF-ERF subfamily, *EVM0018561* was up-regulated while *EVM0000387* and *EVM0019838* were down-regulated, within RPY geng, which suggested that the salt stress tolerance of RPY geng was a very complex process involving many different types of pathways and TFs (Figure 4D).

### Meta-QTLs Mapping of Differentially Expressed Genes Reveals Important Candidate Genes for Salt Stress

**FIGURE 5:**
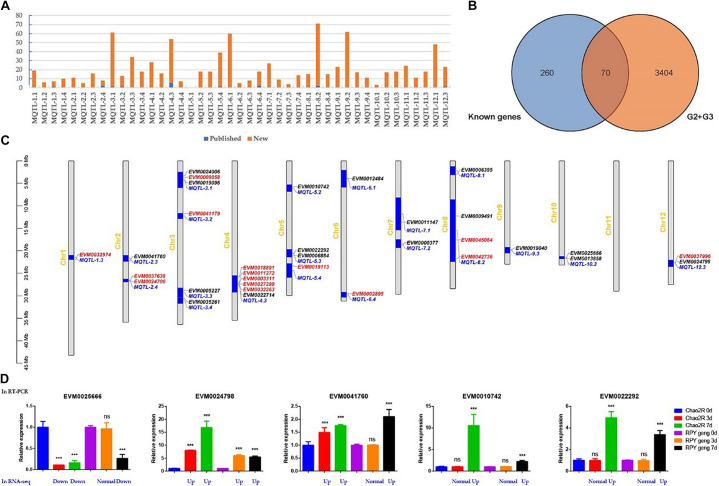
Homologous genes of known genes in Meta-QTLs. **(A)** Statistics of known genes (red) and new genes (blue) of DEGs in each Meta-QTLs. **(B)** The venn analysis result of G2 + G3 and known genes. **(C)** Known genes (red) and homologous genes of known genes (black) in Meta-QTLs. **(D)** RT-PCR and RNA-seq results of five randomly selected candidate genes. G2 + G3 represented RPY geng specific DEGs in panel **(B)**; Red, black, and blue represented known genes, homologous genes of known genes, and Meta-QTLs, respectively; Blue represented gene expression changes relative to the control of RNA-seq in panel **(D)**, details were provided in Supplementary Table 8. ***means that the difference is significant relative to the control (*p* < 0.001), and ns means that the difference is not significant compared to the control.

In order to obtain salt-stress-related candidate genes, we thus collected 43 salt-stress-related Meta-QTLs (Figure 5A and Supplementary Table 4) for Meta-QTLs mapping and 330 salt-stress-related genes for homologous genes identification (Figure 5B and Supplementary Table 5). Here, 42 Meta-QTLs contained varying amounts of DEGs (from G2 + G3) ranging 3 to 71, and 9 Meta-QTLs contained different number of known genes from 1 to 5 (Figure 5A and Supplementary Table 6). Notably, G2 + G3 covered 70 known salt stress-related genes (Figure 5B and Supplementary Table 7), suggesting that the previously reported salt-stress-related genes also played important roles in RPY geng stress tolerance.

We noticed that there were still overfull DEGs among Meta-QTLs after Meta-QTLs mapping, which made it difficult to predict candidate genes. Due to homologous genes often have similar biological functions, we thus searched the homologous genes of 330 known genes in all Meta-QTLs using blastn (Evalue 1E-20). Finally, 18 homologous genes of known genes were obtained in 15 Meta-QTLs, which effectively narrowed the range of candidate genes (Figure 5C and Table 1). We randomly selected five genes for RT-PCR verification. The results of RT-PCR and RNA-seq consistently supported that these genes were salt stress-responsive genes (Figure 5D and Supplementary Table 8).

**TABLE 1:** Eighteen homologous genes of known genes.

**DEG IDs**	**Known gene IDs**	**Meta-QTLs**	**Gene annotations**
EVM0010742	SNAC3	MQTL-5.2	NAC transcription factor
EVM0041760	OsNAC2	MQTL-2.3	NAC transcription factor
EVM0019040	OsNAC6	MQTL-9.3	NAC domain transcription factor
EVM0022292	OsNAC5; OsNAC9; OsNAP; OsNAC6	MQTL-5.3	NAC domain transcription factor
EVM0024006	OsHsfB2b	MQTL-3.1	Heat shock factor
EVM0035261	OsHsfB2b	MQTL-3.4	Heat shock factor
EVM0000377	Osmyb4; OsMYB91	MQTL-7.2	MYB transcription factors
EVM0006395	Osmyb4; OsMYB91	MQTL-8.1	MYB transcription factors
EVM0022714	RCc3	MQTL-4.3	LTPL112 – Protease inhibitor/seed storage/LTP family protein precursor
EVM0025666	RCc3	MQTL-10.3	LTPL112 – Protease inhibitor/seed storage/LTP family protein precursor
EVM0013858	RCc3	MQTL-10.3	LTPL112 – Protease inhibitor/seed storage/LTP family protein precursor
EVM0012484	OsVP1	MQTL-6.1	Inorganic H + pyrophosphatase
EVM0006884	OsKAT1	MQTL-5.3	Potassium channel gene
EVM0024798	RSOsPR10	MQTL-12.3	Pathogenesis-related Bet v I family protein
EVM0009491	OsMGD	MQTL-8.2	Monogalactosyldiacylglycerol synthase gene
EVM0005227	OsGS1;1	MQTL-3.3	Glutamine synthetase
EVM0011147	OsPM19L1	MQTL-7.1	AWPM-19-like membrane family protein
EVM0019096	OsBIERF4; OsEREBP2	MQTL-3.1	AP2 domain containing protein

## Discussion

In view of the catastrophic effects of salt stress on rice, several studies have so far explored the transcriptional changes of rice under salt stress. These earlier studies focused on only a variety or one subspecies (Mizuno et al., 2010; Wang et al., 2018; Mansuri et al., 2019). For example, Nipponbare (*janopinca*) was used in Mizuno et al. (2010) study, Xian156 (*indica*) and IR28 (*indica*) were used in Wang et al. (2018) study, as well as FL478 (*indica*) and IR29 (*indica*) were used in Mansuri et al. (2019) study. However, the difference in salt tolerance between *indica* and *japonica* has not been fully explained, which may be related to the differences in genome and transcription between them. In order to take advantage of the difference between *indica* and *japonica* rice to cultivate new rice varieties with higher salt tolerance by inter-subspecies hybrids, it is necessary to explore the transcriptional differences in the tolerance of *indica* and *japonica* and the underlying molecular mechanisms. Our GO analysis implied that RPY geng had salt stress-related DEGs which conferred higher salt stress tolerance. The DEGs annotation results of these two varieties are completely consistent with the comparative analysis result of the family genes of Chao 2R and RPY geng genomes wherein the RPY geng-specific gene families are closely related to abiotic stress, while the Chao 2R-specific gene families are enriched in essential life activities (unpublished data). Therefore, the results of transcription and genomic data both suggest that the difference in salt stress tolerance between Chao 2R and RPY geng is due to differences in genetic data sets. In the current study, the integrated survival rate and transcription annotation results concluded that RPY geng (*japonica*) has stronger salt stress tolerance than Chao 2R (*indica*). Differently, we reported earlier that TNG67 (*indica*) is more tolerant to salt stress than TCN1 (*japonica*) (Kong et al., 2019b). Lee et al. (2003) also reported the salt tolerance level of *indica* was higher than that of *japonica*. Chen et al. (2018) reported that 9311 (*indica*) has higher salt stress than Nipponbare (*japonica*) and found that the abundance variation of *OsHAK1* transcript underlies the differential salinity tolerance of 9311 and Nipponbare. These results indicated that the difference in salt tolerance between *indica* and *japonica* rice is not absolute, and the difference in salt tolerance may be related to important QTLs, such as the above-mentioned *OsHAK1*. Major QTLs related to salt stress difference between RPY geng and Chao 2R are expected to be fine mapped by combining QTL mapping in the derived populations from a cross of RPY geng and Chao 2R and RNA-seq analysis in immediate future.

Salt stress tolerance is a complicated quantitative trait controlled by multiple genes (Yang et al., 2015; Kong et al., 2019b). In this study, RPY geng (salt-tolerant rice) specific DEGs involved multiple biological pathways closely related to abiotic stress. Previous studies have shown that Ca^2+^-mediated signaling pathways, MAPK, ROS, and ABA signaling pathways played an important role in plant response to salt stress (Tuteja, 2007; Golldack et al., 2014). These signal genes were also significantly enriched in RPY geng specific DEGs, which was in line with the original studies (Tuteja, 2007; Golldack et al., 2014). A large number of novel TFs were identified in this study, suggesting that TFs also played crucial roles in response to salt stress and differential tolerance between *indica* and *japonica* rice. For example, previous studies have shown that WRKY TFs were important regulators of plant salt stress tolerance (Jiang et al., 2017). Another study reported that rice OsMYB2 TF was involved in the salt tolerance, cold tolerance and dehydration tolerance (An et al., 2012). Many phytohormones are important secondary signal molecules that can regulate a variety of external environmental stimuli (Duan et al., 2013). For example, several genes in ABA synthesis and signal transduction pathways can respond to salt stress and increase the ABA content in plants under salt stress (Wang et al., 2014; Shu et al., 2017). In addition, salt stress will reduce the activity level of GA, cause the accumulation of DELLA protein, and then inhibit plant growth to adapt to the stress (Achard et al., 2006). As shown in Figure 4B, these important DEGs involving multiple biological processes may endow RPY geng with higher stress tolerance than Chao 2R and provide potential gene targets for future gene editing verification.

Previous rice salt stress tolerance transcriptome analysis mostly described the GO or KEGG pathway involved in DEGs, but important DEGs closely related to stress tolerance were not focused (Mizuno et al., 2010; Wang et al., 2018; Mansuri et al., 2019). The continuous release of salt tolerance QTL results from different populations has made it possible for us to integrate the transcriptome results to highlight key candidate genes. Here, we innovatively integrated the results of multiple omics including our DEGs, the published Meta-QTLs, as well as known salt stress genes and finally obtained 18 candidate genes, which provided reliable targets for subsequent breeding. Previous studies reported that *OsNAC2* encodes a NAC TF (Mao et al., 2020) and transgenic lines of over-expressing *OsNAC2* gene are more sensitive to high salt stress (Shen et al., 2017). We also found a homologous gene (*EVM0041760*) of *OsNAC2* in MQTL-2.3, and speculated that this gene may also play an important role in rice salt stress tolerance through a molecular regulatory mechanism similar to *OsNAC2*. Additionally, *EVM0022292* in MQTL-5.3 is a homolog of four several known genes belonging to NAC domain transcription factor gene family [*OsNAC5* (Takasaki et al., 2010); *OsNAC9* (Liu et al., 2014); *OsNAP* (Chen et al., 2014); and *OsNAC6* (Hu et al., 2008)], which emphasize the indispensable role of the NAC family in salt stress tolerance. Besides, *OsHsfB2b*, a heat shock factor was considered a core regulator in the defense response to heat stress and negatively regulates the tolerance of rice to drought stress. In this study, *EVM0024006* and *EVM0035261* were identified as homologous genes of *OsHsfB2b* suggesting that heat shock factors may also play an important role in salt stress, and there may be links between different stress regulatory networks. In addition, *EVM0000377* and *EVM0006395* were homologous genes of *Osmyb4* and *OsMYB91*. Overexpression of the rice *Osmyb4* gene enhanced chilling and freezing tolerance in *Arabidopsis* (Vannini et al., 2004) and *OsMYB91* had a function in coordinating plant growth and salt stress tolerance in rice (Zhu et al., 2015). *EVM0022714*, *EVM0025666*, and *EVM0013858* were homologous genes of *RCc3* and *RCc3* has been proven to improve root system architecture and enhance salt tolerance in rice (Li et al., 2018). *OsVP1*, the homologous gene of *EVM0012484*, participated in drought tolerance, salt tolerance, and cold tolerance in rice (Liu et al., 2010; Zhang et al., 2011). *OsKAT1*, the homologous gene of *EVM0006884*, was involved in salt tolerance of rice in cooperation with other K^+^ channels by participating in maintenance of cytosolic cation homeostasis during salt stress, and thus protected cells from Na^+^ (Obata et al., 2007). *RSOsPR10*, the homologous gene of *EVM0024798*, was a novel rice PR10 protein, which was rapidly induced in roots by salt, drought stresses and blast fungus infection possibly through activation of the jasmonic acid signaling pathway (Hashimoto et al., 2004). Another study reported that the expression of *OsMGD* (the homologous gene of *EVM0009491*) was induced by ethephon, gibberellin, drought, and salt treatment, suggesting that *OsMGD* plays a key role in abiotic stress response (Qi et al., 2004). Co-overexpression of *OsGS1;1* (the homologous gene of *EVM0009491*) and *OsGS2* in transgenic rice plants enhanced its tolerance to osmotic and salinity stress at the seedling stage (James et al., 2018). *OsPM19L1*, the homologous gene of *EVM0011147*, encoding *Oryza sativa* AWPM-19-like protein 1 was proven to be dramatically induced by 20% PEG stress, exogenous abscisic acid, salt, and cold stress and associated with stress tolerance through ABA-dependent pathway in rice (Chen et al., 2015). *OsBIERF4* and *OsEREBP2* were homologous genes of *EVM0019096* and have been experimentally proven to be significantly induced by salt stress (Cao et al., 2006; Serra et al., 2013). Overall, our multi-omics analysis results highlighted the important regulatory genes of salt stress in Meta-QTLs. The candidate genes highlighted in this study provide reliable targets for gene editing to produce rice varieties with higher salt tolerance.

## Conclusion

The comparative genomic results of Chao 2R and RPY geng under salt stress indicated that the difference in salt stress tolerance between *indica* and *japonica* rice is most likely due to the difference in the DEGs data set related to salt stress. Here, we further pointed out these genes involved in multiple regulatory pathways in the RPY geng-specific data set can be applied to future gene editing and breeding through multiple annotation methods and multi-omics joint analysis.

## Data Availability Statement

The original contributions presented in the study are publicly available. This data can be found here: National Center for Biotechnology Information (NCBI) BioProject database under accession number PRJNA732136.

## Author Contributions

WK performed all of the experiments, analyzed the data, prepared the figures and tables, and wrote the manuscript. WK and YL conceived and designed the experiments. TS and CZ performed parts of the experiments, figures, and tables. XD provided comments during the writing. All authors read and approved the final version of the manuscript.

## Conflict of Interest

The authors declare that the research was conducted in the absence of any commercial or financial relationships that could be construed as a potential conflict of interest.

## Publisher’s Note

All claims expressed in this article are solely those of the authors and do not necessarily represent those of their affiliated organizations, or those of the publisher, the editors and the reviewers. Any product that may be evaluated in this article, or claim that may be made by its manufacturer, is not guaranteed or endorsed by the publisher.

## Footnotes

1
http://www.plabipd.de/portal/mercator-sequence-annotation

2
http://itak.feilab.net/cgi-bin/itak/index.cgi

3
https://rapdb.dna.affrc.go.jp/tools/blast

## Supplementary Material

Supplementary Figure 1 Schematic diagram of salt stress treatment of samples in this study.

Supplementary Figure 2 The high-resolution figure of Figure 4.

Supplementary Table 1 Sequencing data and sample mapping rates.

Supplementary Table 2 Primers used in RT-PCR.

Supplementary Table 3 Differentially expressed genes (DEGs) in G2 with the opposite expressed profiles in two subspecies.

Supplementary Table 4 Meta-QTLs collected in this study.

Supplementary Table 5 Salt stress-related genes collected in this study.

Supplementary Table 6 RPY geng specific DEGs in Meta-QTLs.

Supplementary Table 7 Seventy known salt stress-related genes in G2 and G3.

Supplementary Table 8 RNA-seq results of five randomly selected DEGs.
