# Supplementary figures and images for: Comparative Transcriptome Analysis Reveals the Mechanisms Underlying Differences in Salt Tolerance Between indica and japonica Rice at Seedling Stage
Source: Front Plant Sci. 2021 Oct 27;12:725436. doi: 10.3389/fpls.2021.725436 (PMC8578091; doi:10.3389/fpls.2021.725436)

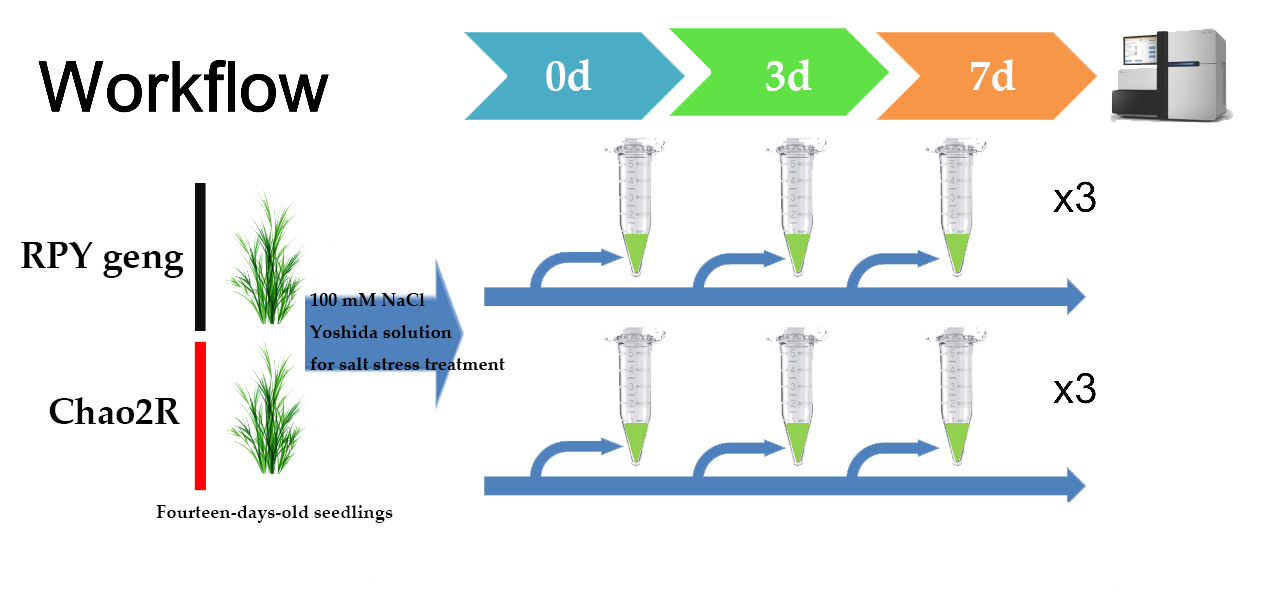


**Figure S1. Schematic diagram of salt stress treatment of samples in this study**

Supplement: Supplementary Figure 1 — Schematic diagram of salt stress treatment of samples in this study. [file Data_Sheet_1.DOCX]
