# Supplementary material for: Comparative Transcriptome Analysis Reveals the Mechanisms Underlying Differences in Salt Tolerance Between indica and japonica Rice at Seedling Stage
Source: Front Plant Sci. 2021 Oct 27;12:725436. doi: 10.3389/fpls.2021.725436 (PMC8578091; doi:10.3389/fpls.2021.725436)
Supplement: Supplementary Figure 2 — The high-resolution figure of Figure 4. [file Data_Sheet_2.PDF]

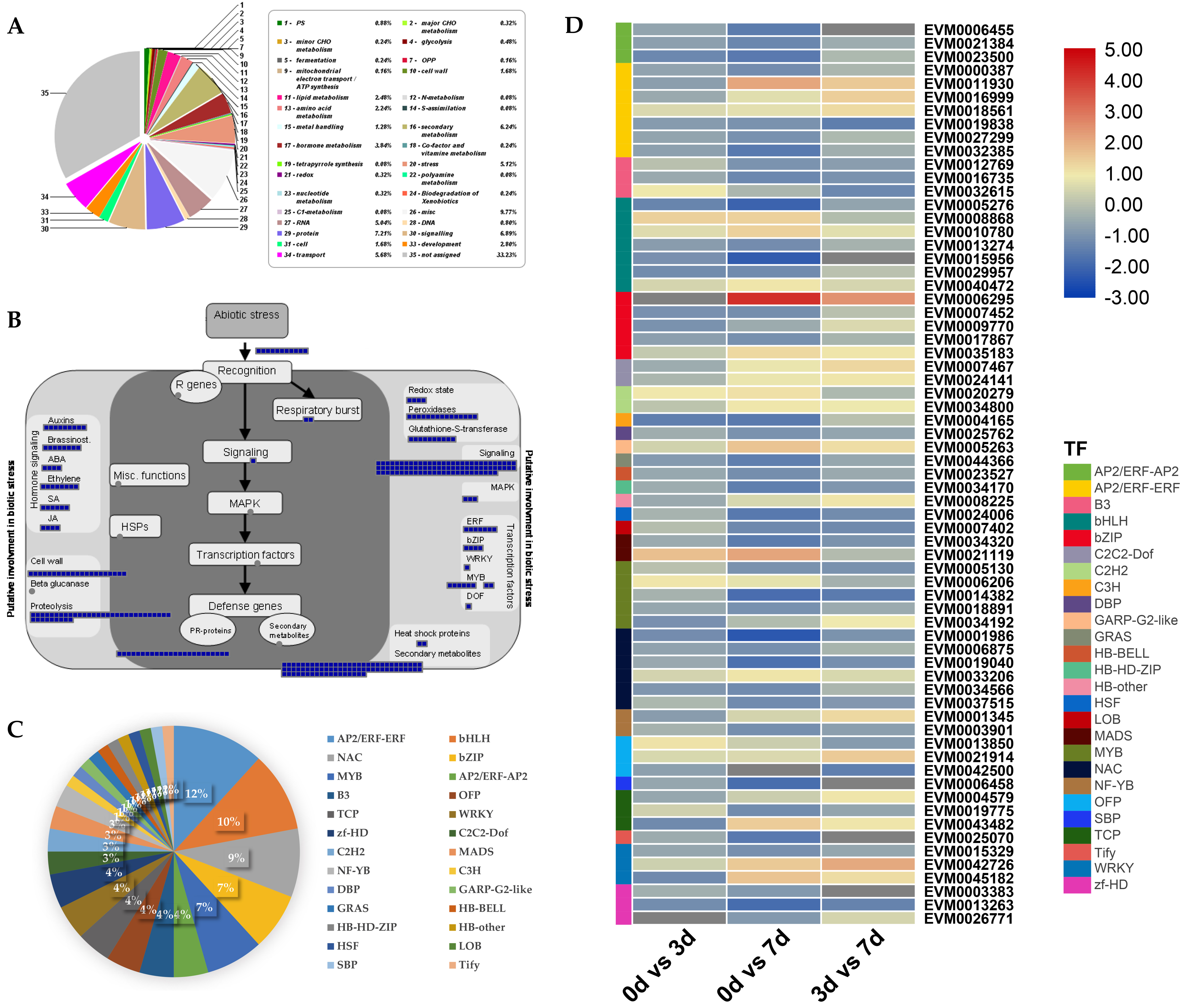

A
1 - PS 0.88%
2 - major CHO metabolism 0.32%
3 - minor CHO metabolism 0.24%
4 - glycolysis 0.48%
5 - fermentation 0.24%
7 - OPP 0.16%
9 - mitochondrial electron transport / ATP synthesis 0.16%
10 - cell wall 1.68%
11 - lipid metabolism 2.48%
12 - N-metabolism 0.08%
13 - amino acid metabolism 2.24%
14 - S-assimilation 0.08%
15 - metal handling 1.28%
16 - secondary metabolism 6.24%
17 - hormone metabolism 3.84%
18 - Co-factor and vitamine metabolism 0.24%
19 - tetrapyrrole synthesis 0.08%
20 - stress 5.12%
21 - redox 0.32%
22 - polyamine metabolism 0.08%
23 - nucleotide metabolism 0.32%
24 - Biodegradation of Xenobiotics 0.24%
25 - C1-metabolism 0.08%
26 - misc 9.77%
27 - RNA 5.04%
28 - DNA 0.80%
29 - protein 7.21%
30 - signalling 6.89%
31 - cell 1.68%
33 - development 2.80%
34 - transport 5.68%
35 - not assigned 33.23%
B
Abiotic stress
Recognition
R genes
Respiratory burst
Signaling
MAPK
Transcription factors
Defense genes
PR-proteins
Secondary metabolites
Misc. functions
HSPs
Hormone signaling
Auxins
Brassinost.
ABA
Ethylene
SA
JA
Cell wall
Beta glucanase
Proteolysis
Redox state
Peroxidases
Glutathione-S-transferase
Signaling
MAPK
ERF
bZIP
WRKY
MYB
DOF
Transcription factors
Heat shock proteins
Secondary metabolites
Putative involvement in biotic stress
Putative involvement in biotic stress
C
12%
10%
9%
7%
7%
4%
4%
4%
4%
4%
3%
3%
3%
AP2/ERF-ERF
bHLH
NAC
bZIP
MYB
AP2/ERF-AP2
B3
OFP
TCP
WRKY
zf-HD
C2C2-Dof
C2H2
MADS
NF-YB
C3H
DBP
GARP-G2-like
GRAS
HB-BELL
HB-HD-ZIP
HB-other
HSF
LOB
SBP
Tify
D
EVM0006455
EVM0021384
EVM0023500
EVM0000387
EVM0011930
EVM0016999
EVM0018561
EVM0019838
EVM0027299
EVM0032385
EVM0012769
EVM0016735
EVM0032615
EVM0005276
EVM0008868
EVM0010780
EVM0013274
EVM0015956
EVM0029957
EVM0040472
EVM0006295
EVM0007452
EVM0009770
EVM0017867
EVM0035183
EVM0007467
EVM0024141
EVM0020279
EVM0034800
EVM0004165
EVM0025762
EVM0005263
EVM0044366
EVM0023527
EVM0034170
EVM0008225
EVM0024006
EVM0007402
EVM0034320
EVM0021119
EVM0005130
EVM0006206
EVM0014382
EVM0018891
EVM0034192
EVM0001986
EVM0006875
EVM0019040
EVM0033206
EVM0034566
EVM0037515
EVM0001345
EVM0003901
EVM0013850
EVM0021914
EVM0042500
EVM0006458
EVM0004579
EVM0019775
EVM0043482
EVM0025070
EVM0015329
EVM0042726
EVM0045182
EVM0003383
EVM0013263
EVM0026771
0d vs 3d
0d vs 7d
3d vs 7d
5.00
4.00
3.00
2.00
1.00
0.00
-1.00
-2.00
-3.00
TF
AP2/ERF-AP2
AP2/ERF-ERF
B3
bHLH
bZIP
C2C2-Dof
C2H2
C3H
DBP
GARP-G2-like
GRAS
HB-BELL
HB-HD-ZIP
HB-other
HSF
LOB
MADS
MYB
NAC
NF-YB
OFP
SBP
TCP
Tify
WRKY
zf-HD
